# Estimating Conformational Traits in Dairy Cattle With DeepAPS: A Two-Step Deep Learning Automated Phenotyping and Segmentation Approach

**DOI:** 10.3389/fgene.2020.00513

**Published:** 2020-05-21

**Authors:** Jessica Nye, Laura M. Zingaretti, Miguel Pérez-Enciso

**Affiliations:** ^1^Centre for Research in Agricultural Genomics (CRAG), CSIC-IRTA-UAB-UB Consortium, Barcelona, Spain; ^2^ICREA, Barcelona, Spain

**Keywords:** image analysis, morphology, phenomics, image mask, deep learning, dairy cattle

## Abstract

Assessing conformation features in an accurate and rapid manner remains a challenge in the dairy industry. While recent developments in computer vision has greatly improved automated background removal, these methods have not been fully translated to biological studies. Here, we present a composite method (DeepAPS) that combines two readily available algorithms in order to create a precise mask for an animal image. This method performs accurately when compared with manual classification of proportion of coat color with an adjusted *R*^2^ = 0.926. Using the output mask, we are able to automatically extract useful phenotypic information for 14 additional morphological features. Using pedigree and image information from a web catalog (www.semex.com), we estimated high heritabilities (ranging from *h*^2^ = 0.18–0.82), indicating that meaningful biological information has been extracted automatically from imaging data. This method can be applied to other datasets and requires only a minimal number of image annotations (∼50) to train this partially supervised machine-learning approach. DeepAPS allows for the rapid and accurate quantification of multiple phenotypic measurements while minimizing study cost. The pipeline is available at https://github.com/lauzingaretti/deepaps.

## Introduction

Breeding programs depend on large-scale, accurate phenotyping, which is also critical for genomic dissection of complex traits. While the genome of an organism can be characterized, e.g., with high density genotyping arrays, the “phenome” is much more complex and can never be fully described, as it varies over time and changes with the environment (Houle et al., 2010). The cost of genotyping continues to drop, but there is still a need for improvements in obtaining high-performance phenotypes at a lower cost (Tardieu et al., 2017). In cattle, the number of phenotypes recorded in traditional breeding schemes is relatively small, because its recording is expensive. For instance, yearly milk yield is usually inferred by extrapolation using a few lactation measurements, whereas actual milk production can now be measured individually and daily using automated milking robots.

In addition to milk yield, dairy cattle breeders are interested in conformational traits. These metrics are not only relevant aesthetically but can also have an important influence on an animal’s breeding value. Body conformation is associated with dairy performance (Guliński et al., 2005) and longevity, which strongly contributes to lifetime milk production (Sawa et al., 2013). Milk production is positively correlated with udder size (Mingoas et al., 2017). The highest negative economic impact for dairy farmers is caused by lameness either due to leg malformations or injury (Sogstad et al., 2006; Green et al., 2010). Extracting the detailed conformational phenotypes which may impact progeny success are likewise time consuming and costly to collect, and in the absence of quantitative tools, farmers often evaluate morphometric measurements qualitatively.

The emergence of modern sensor technologies, such as Unmanned Aerial Vehicles (UAV) combined with simple digital cameras (Kefauver et al., 2017), mass spectroscopy, robotics, and hyper-spectral images (Fahlgren et al., 2015), among others, have revolutionized breeding programs, mainly in plants, allowing for non-invasive evaluation of multiple complex traits. Although in animal breeding their application is more scarce, modern livestock farming is beginning to benefit from access to these inexpensive sensor tools. Now, it is possible to remotely monitor behavior (Guzhva et al., 2016; Foris et al., 2019; Zehner et al., 2019) and animal welfare (Beer et al., 2016), assess movement (Chapinal et al., 2011), measure body confirmation (Van Hertem et al., 2013; Song et al., 2018), quantify individual food intake (Braun et al., 2014; Beer et al., 2016; Foris et al., 2019), maintain an optimum environment (Chen and Chen, 2019), or decrease instances of stillbirths (Palombi et al., 2013; Ouellet et al., 2016). These automated measurements rely on temperature (Palombi et al., 2013; Ouellet et al., 2016; Chen and Chen, 2019), pressure (Braun et al., 2014; Beer et al., 2016), movement (Chapinal et al., 2011), and visual (Van Hertem et al., 2013; Guzhva et al., 2016; Song et al., 2018; Foris et al., 2019; Zehner et al., 2019) sensors.

As several remote monitoring schemes are based on digital images or video, automated image analysis techniques are urgently needed to quantify traits of interest (Zhang et al., 2018). Applying image analysis to breeding programs is not new, however many of these methods largely depend on time consuming image-by-image processing facilitated by the researcher (as in Hayes et al., 2010; Cortes et al., 2017; Rosero et al., 2019). The few automated resources currently implemented for cattle analyses require complicated set-ups and costly equipment (Chapinal et al., 2011; Song et al., 2018). This is not surprising as accurately quantifying phenotypic information is one of the most challenging aspects in biology (Houle et al., 2011; Boggess et al., 2013; Rahaman et al., 2015).

The availability of new algorithms based on machine learning has revolutionized computer vision, impacting a wide range of fields that rely on computers to analyze images, with the potential to optimize herd care and improve animal and plant breeding program outcomes (Song et al., 2018; Foris et al., 2019; Zehner et al., 2019). These recent advances have led to precise object detection and semantic segmentation in complex images (Girshick et al., 2014; Han et al., 2018; Gu et al., 2018).

Here, we show how automatically parsed web-based catalog datasets can be converted into useful information by automatically inferring genetic parameters of several morphological measurements in dairy cattle. We combined web scraping, deep learning, and statistical techniques in order to achieve our objective. The proposed methodology is a mixture between a supervised deep learning approach, Mask R-CNN (He et al., 2017) and an unsupervised algorithm (Kanezaki, 2018) which can achieve highly precise automatic image segmentation. After removing the background, phenotypic information, including coat color and body conformational traits can be easily quantified. Lastly, we demonstrate the potential applications of this method in other datasets. We assert that our work could constitute a good proxy for using inexpensive and non-invasive computer vision techniques into the dairy cattle breeding programs.

## Materials and Methods

### Image Collection

Images of bulls were collected through web-scraping using the python library Beautiful Soup (Richardson, 2007). Images from sire catalogs of six Artificial Insemination companies were collected. We additionally automatically collected bull images from one semen provider^1^ and those of identified familial relationships (daughters, dams, granddams, and great granddams) where possible. We downloaded a total of 1,819 images. These images ranged in size between 339–879 pixels and 257–672 pixels for width and height, respectively. The animals are Holstein with patched black and white bodies, but some images are red Holstein. Individuals ranged in color from all white, all black, all brown, to a mixture of the colors. The images were flipped so that all animals faced the right side of the image using ImageMagick version 7.0.9-0 convert -flop function. The animals are standing in front of dynamic backgrounds including forest, field, snow, water, and straw. All images contained only one animal, and sometimes contained a person or an arm.

### Automated Segmentation

One of the most challenging tasks in computer vision is instance segmentation, i.e., the identification of boundaries of objects at the pixel level (Kanezaki, 2018), whereas object classification, i.e., to determine if an object belongs to certain class is relatively simpler. R-CNN (Girshick et al., 2014), a deep learning approach, as well as Fast R-CNN (Girshick, 2015), Faster R-CNN (Ren et al., 2015), or Mask R-CNN (He et al., 2017) are widely used to solve this task. Although these methods are efficient, they are not accurate enough for some purposes since the obtained segmentation often removes parts of the object of interest or contains parts of the background.

**FIGURE 1:**
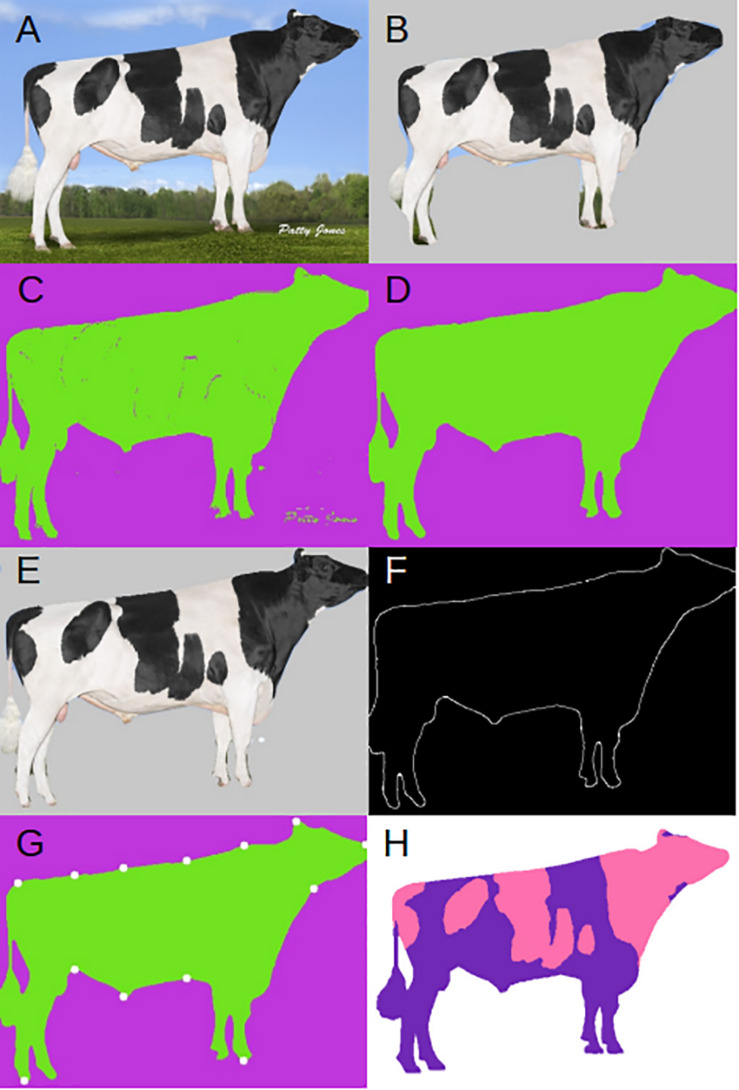
Example input and outputs. **(A)** Original input image. **(B)** Mask R-CNN applied mask. **(C)** DeepAPS raw output. **(D)** Final output of DeepAPS after all applied filters. **(E)** Final DeepAPS mask applied to input image. **(F)** Outline extraction of original input image. **(G)** Extracted landmark coordinates. **(H)** Manual color segmentation. Image from Semex.

We applied a two-step procedure to automatically segment the animal’s profile as accurately as possible. The composite method begins by using Mask R-CNN (He et al., 2017), which has three outputs for each candidate object in an input image (Figure 1A): a class label (say “cow”), bounding box offset or region of interest (RoI), and the object mask consisting of an approximate layout of a spatial object. As in the original Mask R-CNN, we used the annotated image database common objects in context (COCO; Lin et al., 2014)^2^ to train the algorithm, and select the class codes for cow. In short, Mask R-CNN is a deep learning algorithm that consists of two steps: first, it proposes regions within the image that may contain objects of interest and, second, generates a mask for every detected object. The latter step consists of a binary classification of pixels, either a pixel belongs to the object or to the background. For more details about this method readers can consult, e.g., https://towardsdatascience.com/computer-vision-instance-segmentation-with-mask-r-cnn-7983502fcad1, https://engineering.matterport.com/splash-of-color-instance-segmentation-with-mask-r-cnn-and-tensorflow-7c761e238b46 or should refer to He et al. (2017). Figure 1B shows the applied mask predicted by Mask R-CNN, this mask removes the majority of the background, but also removes parts of the cow’s body making it necessary for the development of our two-step composite method. We used the implementation of Mask R-CNN in https://github.com/matterport/Mask_RCNN.

After the RoI and class labels are extracted, we select only the RoI for our desired object (i.e., the bull or cow). This allows us to remove some of the background and obtain a smaller, less noisy image. As explained above, the Mask R-CNN segmentation was not accurate enough for our purposes (Figure 1B). Therefore, we passed the RoI and predicted mask to a modified version of the unsupervised image segmentation algorithm from Kanezaki (2018). We used the code available at https://github.com/kanezaki/pytorch-unsupervised-segmentation. The original algorithm relies on separating pixels from each other and grouping them into distinct clusters based on color and texture. The underlying assumptions of this model are that: (1) pixels of similar features should be clustered together, (2) spatially continuous pixels should be clustered together, and (3) the number of clusters should be large. This is achieved by applying a linear classifier which groups pixels into different clusters based on their features. The difference between the original algorithm and ours is we do not try to maximize the total number of clusters, but instead we merely improve upon the mask generated by Mask R-CNN based on pixel identity. This makes more effective the algorithm to run, since the algorithm applied to the whole original image was not completely satisfactory. This proceeds by self-training the network through back propagation, by alternating between two stages: (1) forward super pixel refinement, and (2) backward gradient descent. Much like any supervised approach this is achieved by calculating the cross-entropy loss between network and cluster labels, then back propagating the error rates used to update the convolutional filter parameters. Backpropagation is a popular and clever method used in deep learning. It allows computing the gradient of the loss function very efficiently by using the chain rule for derivatives, which greatly simplifies optimization in complex models.

After refinement through the unsupervised algorithm, we obtained a relatively precise mask for our input image (Figure 1C). However, the unsupervised clustering still can confound the foreground and the background. We then apply an additional filter to the mask, median blur function from OpenCV (Bradski, 2000), removing small islands that have been mislabeled during the clustering step (Figure 1D). We lastly apply the mask by coloring all pixels predicted to be in the background by a solid color (Figure 1E).

To extract the proportion of and average color(s) from each cluster, we apply k-means using the scikit-learn library (Pedregosa et al., 2011). To measure anatomical features, we extract only the outline of the desired object from the mask (Figure 1F) using the edge detection algorithm developed by Canny (1986) and implemented in OpenCV (Bradski, 2000). After extracting the edge, we apply one more filter to remove any islands that may remain using the remove_small_objects function from the morphology package available from scikit-learn (Pedregosa et al., 2011). Now that the input image has been reduced down to just the object outline, we can take advantage of common conformational features of the underlying data, and extract pixel coordinates. For example, we extracted the coordinate of the pixel closest to the bottom left corner which corresponds to the back foot of the cow. We proceeded in this way to extract 13 coordinates from each animal (Figure 1G). We then calculate the distance in pixels between various points, effectively extracting body confirmations automatically. The 14 conformational traits are described in Supplementary Figure S1. Code for the whole pipeline is available at https://github.com/lauzingaretti/deepaps.

### Manual Segmentation

To check how accurate the automated segmentation was, we manually segmented *N* = 481 images that were not of Semex origin. We used Kanezaki’s demo. py program (2018) in python3.6 (van Rossum, 1995) using default parameters. The output images were opened in the image processing software GIMP^3^, and the background was manually changed from the colored cluster to white (Figure 1H). To extract the color clusters, we calculated the proportion of color clusters in each image by using k-means as above, and manually matched each color cluster to the original picture and removed the proportion of background.

### Genetic Parameters

To calculate heritabilities for the measured phenotypes, we extracted pedigree information and constructed a relationship matrix for each bull whenever possible. This was done by automatic web scraping in the sire catalog website, where we identified bull id, any relative type (i.e., daughter, dam, granddam, and great granddam), and their images. From the list of bull and relatives’ ids, we computed the standard numerator relationship matrix, which contains the genetic relationships assuming an infinitesimal model. Bayesian estimates of heritability were calculated with the R 3.5.2. (R Core Team, 2013) package BGLR (Perez and de los Campos, 2014) using default priors. One thousand Gibbs iterations were performed. Our sample sizes were *N* = 1,338 for proportion of white and *N* = 1,062 for morphological characteristics. The difference in sample size is due to removing any image with a missing coordinate.

### Application to Other Datasets

To assess the applicability to other datasets, we chose two other objects that had been annotated in the COCO database (Lin et al., 2014), horse and giraffe, as well as two objects that had not been annotated, butterfly and duck. We downloaded 50 images from the internet that had the license set to “labeled for non-commercial reuse” for horse and giraffe and 100 images for butterfly and duck. For the unannotated objects we annotated 50 of the images using VGG Image Annotator (VIA; Dutta and Zissermann, 2019). These annotations were used to train a model in Mask R-CNN using the starting weights of the COCO database (Lin et al., 2014). The model was trained for 20 epochs and default parameters. Using either the COCO or custom model, DeepAPS was applied and the composite mask was visually assessed for accuracy.

## Results

**FIGURE 2:**
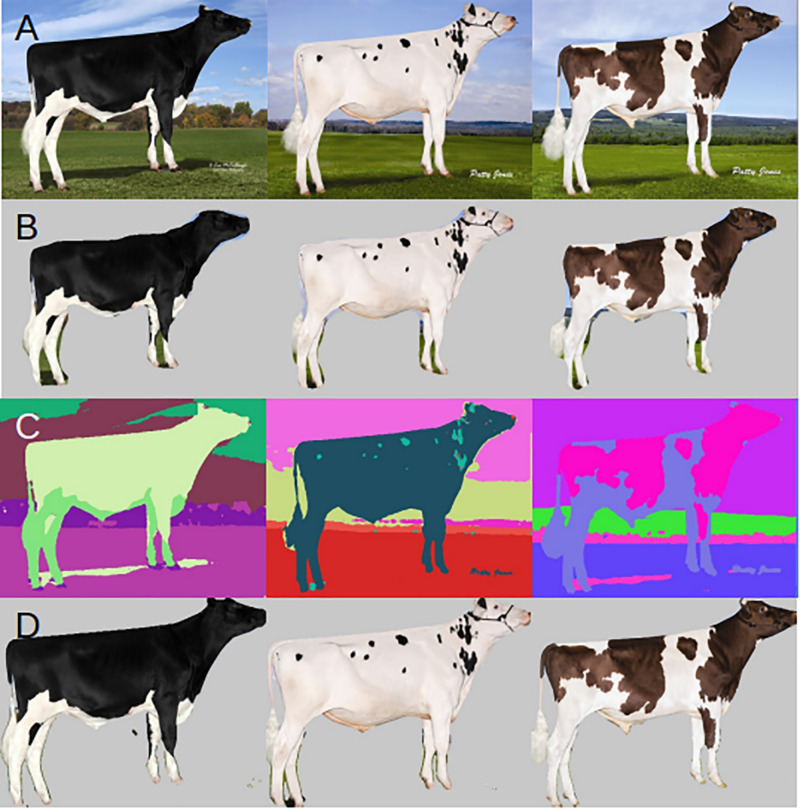
Example input and outputs. **(A)** Original input image. **(B)** Mask R-CNN applied mask. **(C)** DeepAPS raw output. **(D)** Final output of DeepAPS after all applied filters.

We first visually compared the masks generated by the three methods that were applied to our entire dataset of 1,819 images (Figure 2A). When we used the supervised algorithm Mask R-CNN and applied the mask to the input images (Figure 2B), we observed in all cases parts of the cow body were removed along with the background (i.e., tail, nose, ear, and hoof). These masks are not satisfactorily precise to extract morphological measurements. The unsupervised segmentation by back propagation (Figure 2C) often separates the precise border between cow and background, but that this method on its own is not automated. Each output image would still need to be processed separately in order to match which body parts were grouped into each color cluster. DeepAPS (Figure 2D) across our input dataset produces a more accurate mask than Mask R-CNN and a fully automated mask, which the unsupervised approach fails to do.

**FIGURE 3:**
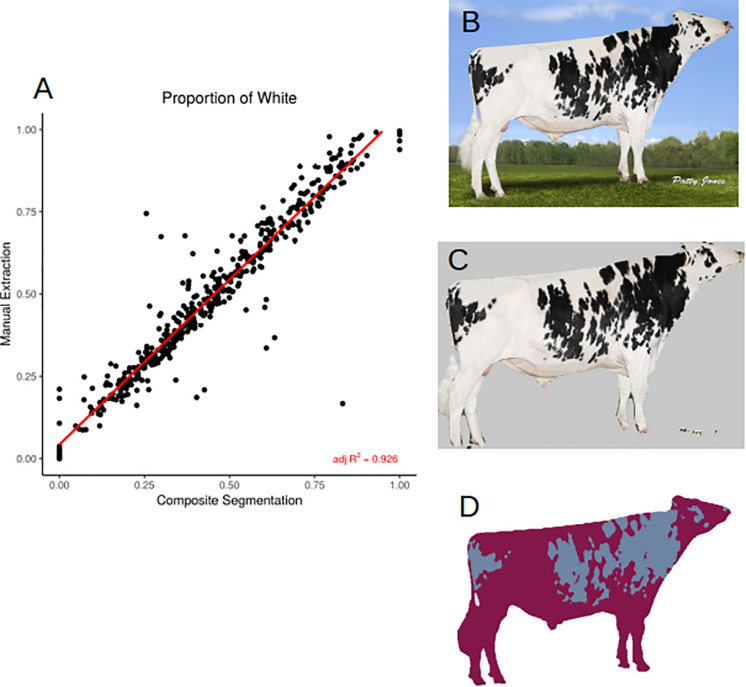
**(A)** Correlation (adjusted *R*^2^ = 0.926) between manual and automated color segmentation of 481 images. **(B)** Example input image. **(C)** Applied DeepAPS output mask. **(D)** Manual color segmentation. Image from Semex.

In order to assess how accurately we were able to extract the true coat color percentage from each image, we compared manual and automated color segmentation. Our test set consists of 481 manually annotated images. After removing the background, we clustered each bull into one- or two-color components and extracted the percentage of dark and light colors in the coats. The automated method reports a highly accurate color segmentation with an adjusted *R*^2^ = 0.926 (Figure 3A) when compared to manual segmentation (Figures 3B–D). The images that fall out as outliers belong to one of two groups, the majority of the outliers have small image sizes (less than 400 × 400 pixels), and therefore the quality was not sufficient to accurately separate the body into two color classes, the second group were bulls with a two-toned body color, in which the legs were of a different color than the body. In these cases, the algorithm has difficulty in separating the dark-colored legs from the dark background.

**FIGURE 4:**
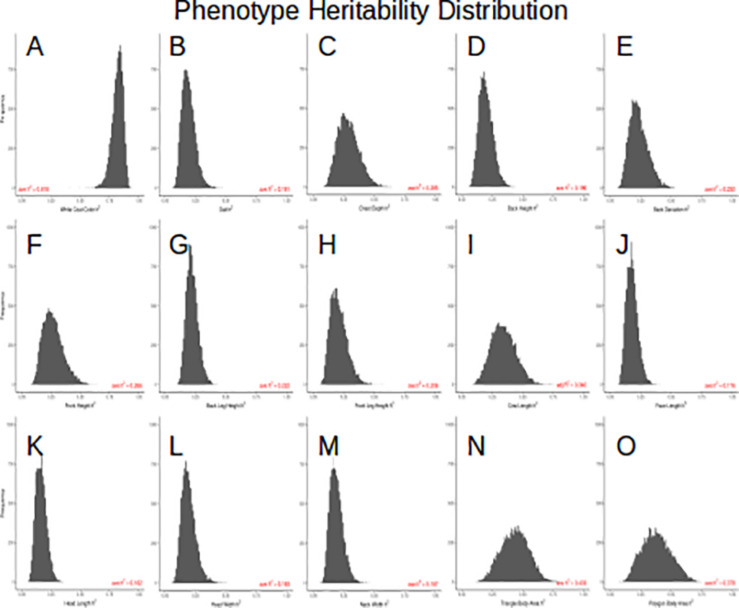
**(A)** posterior *h*^2^ distribution of the white coat color, **(B)** posterior *h*^2^ distribution of the gait, **(C)** posterior *h*^2^ distribution of the chest depth, **(D)** posterior *h*^2^ distribution of the back height, **(E)** posterior *h*^2^ distribution of the back deviation, **(F)** posterior *h*^2^ distribution of the front height, **(G)** posterior *h*^2^ distribution of the back leg height, **(H)** posterior *h*^2^ distribution of the front leg height, **(I)** posterior *h*^2^ distribution of the cow length, **(J)** posterior *h*^2^ distribution of the face length, **(K)** posterior *h*^2^ distribution of the head length, **(L)** posterior *h*^2^ distribution of the head width, **(M)** posterior *h*^2^ distribution of the neck width, **(N)** posterior *h*^2^ distribution of the triangle body area, **(O)** posterior *h*^2^ distribution of the polygon body area.

Because the mask recovered after using this composite method is so precise, we could extract coordinates of 13 points located around the outline of the cow body (Figure 1G and Supplementary Figure S1) which allowed for measurements of 14 body conformation distances (see Supplementary Figure S2 for phenotypic distributions). Next, we estimated heritability using 1,338 images of related animals, in which we had partial information about great granddam, granddam, dam, bull, and daughter relationships. Our relationship matrix consists of 689 families, with an average of 2.6 individuals per family. Figure 4 shows the 15 posterior distributions of the heritability calculations and lists average values. Coat color proportion has the highest calculated heritability *h*^2^ = 0.82, followed by body area (triangle) *h*^2^ = 0.43, body area (polygon) *h*^2^ = 0.38, and cow body length *h*^2^ = 0.34. These values are similar to previously published results (Hayes et al., 2010; Pritchard et al., 2013). These high heritability measurements indicate foremost that the meaningful genetic information can be quickly and easily extracted from imaging and pedigree data available online.

**FIGURE 5:**
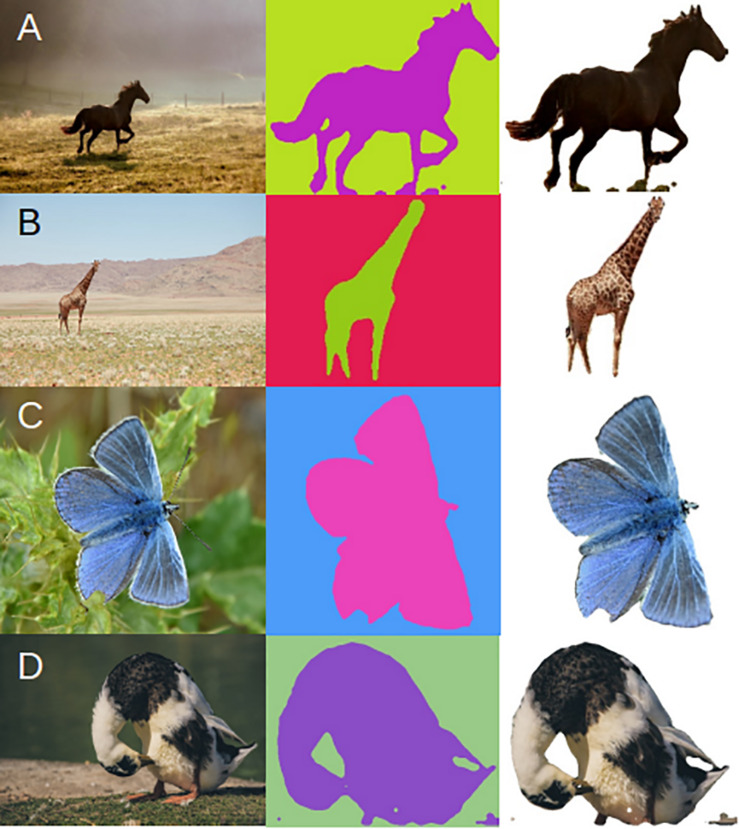
Application of DeepAPS method to four additional datasets. **(A)** Horses and **(B)** Giraffes trained using the COCO database. **(C)** Butterflies and **(D)** Ducks trained using 50 custom annotations.

To assess whether this method is robust to the type and quality of the underlying data, we downloaded images from the internet of horse, giraffe, butterfly, and duck. These images were randomly collected, and we had no control over quality, size, lighting, or background. We also wanted to test how many input annotations are required to produce a robust mask using DeepAPS. Because the two-step method uses back propagation in order to refine the predicted mask generated from the machine learning algorithm, we hypothesized that fewer annotations would be needed. Therefore, we annotated 50 images for the butterfly and duck datasets, as they were not pre-annotated in the COCO database. We found that overall, our composite method preforms accurately (Figure 5). The masks generated from the thousands of annotations from the COCO dataset were precise (Figures 5A,B), while those based on only 50 annotations were still far more accurate than using any currently available method (Figures 5C,D). These results together indicate this method is robust to input data and can still preform reliably despite being trained by few instances, making it a promising tool for automatic morphological analyses.

## Discussion

In recent decades, there have been vast improvements in molecular and statistical methods applied to animal and plant breeding. While modern livestock studies typically involve the analysis of entire genomes and/or vast number of polymorphic sites (Börner et al., 2012; Wiggans et al., 2017; Yin and König, 2019), high throughput phenotyping is lagging, especially in animal breeding. Often, phenotypic variation is explored today in the same manner as it was done decades ago, using simple quantifications such as length, number, categorical classifications, etc (Houle et al., 2010, 2011; Cole et al., 2011). Phenomics is extremely important in breeding programs in particular, as the desired outcome is a change in a phenotype. As phenotypes are formed by a complex process involving multiple genes, is dependent on the environment, and dynamic overtime, collecting multiple descriptive statistics can make relating genotype to phenotype more feasible and, importantly, more meaningful.

Images are among the easiest to collect data and are underutilized. Here we combine two of the state-of-the-art image analysis tools, the supervised Mask R-CNN (He et al., 2017) and unsupervised segmentation (Kanezaki, 2018) in order to automatically extract phenotypic measurements accurately. Not only can we create a precision mask but can cluster and segment the underlying colors and automatically measure body confirmation. Accurate image segmentation remains the most challenging part of computer vision. The ability of DeepAPS to separate the animal from multiple background types at the pixel level out preforms, for our purposes, the available algorithms currently published (Kanezaki, 2018; He et al., 2017).

The validity and speed of this method allows for multiple quantitative morphological traits to be implemented in breeding programs. Despite the success of ongoing dairy breeding programs (Wiggans et al., 2017), including more and accurately quantified measurements has the potential to result in further improvements (Goddard, 2009; Gonzalez-Recio et al., 2014). Furthermore, this method uses standard side-view stud images which are inexpensive to generate and store. Our presented method eliminates the high cost of phenotype collection while maintaining quality and can contribute to lowering the cost of conformational measurement collections.

Our analyses were performed on images scrubbed from the internet. As such, we had no control over backgrounds, lighting, image size, or quality. Despite the dynamic input data on which we tested DeepAPS, we were able to produce high quality masks and phenotypic measurements in most cases (Figure 4). Furthermore, the heritability rates we calculated from over 1,000 images of related individuals broadly agree with published results, indicating that our method accurately captures underlying information. Hayes et al., 2010) estimated heritability of coat color percentage by manual quantification and reported a heritability of *h*^2^ = 0.74 in *N* = 327 bulls; remarkably, we found similar estimates (*h*^2^ = 0.81), even if our pedigree information was quite incomplete. The reported heritability of back leg height is nearly identical to previous reports (*h*^2^ = 0.22 vs. 0.21; Pritchard et al., 2013). Nevertheless, estimates of two other reported conformational heritabilities were somewhat lower: chest depth *h*^2^ = 0.28 vs. 0.37 and height *h*^2^ = 0.27 vs. 0.42 (Pritchard et al., 2013); perhaps because actual metrics analyzed here are not exactly those used in previous studies and because we cannot obtain absolute values (e.g., height in meters), since there is not a common scale across images. In all, this proof of concept shows how genetic parameters could be estimated using solely data that are already available on the web. For practical applications, more accurate estimates suitable for breeding programs could be obtained, e.g., combining SNP genotyping data with automatic image analyses from larger datasets.

While imaging data is fast and simple to collect as well as inexpensive to store, the most burdensome stage of image analysis is the generation of image annotations. We found that this method is able to leverage the publicly available COCO database and apply it to new and different problem sets. Allowing for the creation of an accurate object mask based only on a training set of 50 instances (Figure 5), which is remarkably low for any machine learning approach.

This method has the potential to allow for imaging data to be easily and quickly applied to high-throughput studies, which can be highly useful and improve extant breeding programs. We provide a combined deep learning algorithm that results in highly accurate segmentation of animal profiles, which is necessary for further processing in applications related to conformational measurements. Nevertheless, we are well aware that much work remains to be done in the area. For instance, software to accurately quantify a number of additional conformational features, such as udder metrics or movement, using different angle pictures or videos should be developed. Software should also be optimized for speed and be able to analyze high-resolution pictures.

## Data Availability Statement

The datasets generated for this study are available on request to the corresponding author.

## Author Contributions

MP-E conceived and supervised the research. JN and LZ performed the research and wrote the code. JN drafted the manuscript with contributions from the other authors.

## Conflict of Interest

The authors declare that the research was conducted in the absence of any commercial or financial relationships that could be construed as a potential conflict of interest.

## Footnotes

1 www.semex.com

2 http://cocodataset.org

3 https://www.gimp.org/

## Supplementary Material

FIGURE S1 Description of the 14 extracted conformational traits.

FIGURE S2 Phenotypic distributions for the 14 measured anatomical features (*N* = 1,062).

## References

[B1] Beer G. Alsaaod M. Starke M. Schuepbach-Regula G. Müller H. Kohler P. (2016). Use of extended characteristics of locomotion and feeding behavior for automated identification of lame dairy cows. *PLoS One* 11:e0155796 10.1371/journal.pone.0155796 PMC4871330 27187073

[B2] Boggess M. V. Lippolis J. D. Hurkman W. J. Fagerquist C. K. Briggs S. P. Gomes A. V. (2013). The need for agriculture phenotyping: “Moving from genotype to phenotype”. *J. Proteo.* 93 20–39. 10.1016/j.jprot.2013.03.021 23563084

[B3] Börner V. Teuscher F. Reinsch N. (2012). Optimum multistage genomic selection in dairy cattle. *J. Dairy Sci.* 95 2097–2107. 10.3168/jds.2011-4381 22459855

[B4] Bradski G. (2000). The OpenCV library. *Dr. Dobbs J.* 120 122–125.

[B5] Braun U. Tschoner T. Hässig M. (2014). Evaluation of eating and rumination behavior using a noseband pressure sensor in cows during the peripartum period. *BMC Vet. Res.* 10:195 10.1186/s12917-014-0195-6 PMC4158097 25203524

[B6] Canny J. (1986). A computational approach to edge detection. *IEEE T. Pattern Anal.* 8 679–698. 21869365

[B7] Chapinal N. de Passillé A. M. Pastell M. Hänninen L. Munksgaard L. Rushen J. (2011). Measurement of acceleration while walking as an automated method for gait assessment in dairy cattle. *J. Dairy Sci.* 94 2895–2901. 10.3168/jds.2010-3882 21605759

[B8] Chen C. S. Chen W. C. (2019). Research and development of automatic monitoring system for livestock farms. *Appl. Sci.* 9:1132.

[B9] Cole J. B. Wiggans G. R. Ma L. Sonstegard T. S. Lawlor T. J. Jr. Crooker B. A. (2011). Genome-wide association analysis of thirty-one production, health, reproduction and body conformation traits in contemporary U.S. Holstein cows. *BMC Genomics* 12:408 10.1186/1471-2164-12-408 PMC3176260 21831322

[B10] Cortes D. F. M. Catarina R. S. Barros G. B. D. A. Arêdes F. A. S. da Silveira S. F. Ferreguetti G. A. (2017). Model-assisted phenotyping by digital images in papaya breeding programs. *Sci. Agric.* 74 294–302.

[B11] Dutta A. Zissermann A. (2019). *The VIA Annotation Software for Images, Audio, and Video.* New York, NY: ACM.

[B12] Fahlgren N. Gehan M. A. Baxter I. (2015). Lights, camera, action: high-throughput plant phenotyping is ready for a close-up. *Curr. Opin. Plant Biol.* 24 93–99. 10.1016/j.pbi.2015.02.006 25733069

[B13] Foris B. Thompson A. J. von Keyserlingk M. A. G. Melzer N. Weary D. M. (2019). Automatic detection of feeding- and drinking-related agonistic behavior and dominance in dairy cows. *J. Dairy Sci.* 102 9176–9186. 10.3168/jds.2019-16697 31400897

[B14] Girshick R. G. (2015). Fast R-CNN. *IEEE Comput. Soc. Conf. Comput. Vis.* 2015 1440–1448.

[B15] Girshick R. G. Donahue J. Darrell T. Malik J. (2014). “Rich feature hierarchies for accurate object detection and semantic segmentation,” in *Proceedings of the 2014 IEEE Conference on Computer Vision and Pattern Recognition*, Columbus, OH, 580–587.

[B16] Goddard M. (2009). Genomic selection: prediction of accuracy and maximization of long-term response. *Genetica* 136 245–257. 10.1007/s10709-008-9308-0 18704696

[B17] Gonzalez-Recio O. Coffey M. P. Pryce J. E. (2014). On the value of the phenotypes in the genomic era. *J. Dairy Sci.* 97 7905–7915. 10.3168/jds.2019-102-6-5764 25453600

[B18] Green L. E. Borkert J. Monti G. Tadich N. (2010). Assocications between lesion-specific lameness and the milk yield of 1,635 dairy cows from seven herds in the Xth region of Chile and implications for management of lame dairy cows worldwide. *Anim. Welfare* 19 419–427.

[B19] Gu J. Wang Z. Kuen J. Ma L. Shahroudy A. Shuai B. (2018). Recent advances in convolutional neural networks. *Pat. Recognit.* 77 354–377.

[B20] Guliński P. Młynek K. Litwiñczuk Z. Dobrogowska E. (2005). Heritabilities of genetic and phenotypic correlations between condition score and production and conformation traits in Black-and-White cows. *Anim. Sci. Pap. Rep.* 23 33–41.

[B21] Guzhva O. Ardö H. Herlin A. Nilsson M. Åström K. Bergsten C. (2016). Feasibility study for the implementation of an automatic system for the detection of social interactions in the waiting area of automatic milking stations by using a video surveillance system. *Comput. Elect. Agric.* 127 506–509.

[B22] Han J. Zhang D. Cheng G. Liu N. Xu D. (2018). Advanced Deep-Learning techniques for salient and category-specific object detection: a survey. *IEEE Signal Process.* 35 84–100.

[B23] Hayes B. J. Pryce J. Chamerlain A. J. Bowman P. J. Goddard M. E. (2010). Genetic architecture of complex traits and accuracy of genomic prediction: coat colour, milk-fat percentage, and type in Holstein Cattle as contrasting model traits. *PLoS Genet.* 6:e1001139 10.1371/journal.pgen.1001139 PMC2944788 20927186

[B24] He K. Gkioxari G. Dollár P. Girshick R. (2017). Mask R-CNN. *IEEE Int. Conf. Comput. Vis.* 2017 2980–2988.

[B25] Houle D. Govindaraju D. R. Omholt S. (2010). Phenomics: the next challenge. *Nat. Rev. Genet.* 11 855–866. 21085204 10.1038/nrg2897

[B26] Houle D. Pélabon C. Wagner G. P. (2011). Measurement and meaning in biology. *Q. Rev. Biol.* 86 3–34. 21495498 10.1086/658408

[B27] Kanezaki A. (2018). Unsupervised image segmentation by backpropagation. *IEEE Int. Conf. Comput. Vis.* 1543–1547.

[B28] Kefauver S. C. Vincente R. Vergara-Díaz O. Fernandez-Gallego J. A. Kerfal S. Lopez A. (2017). Comparative UAV and field phenotyping to assess yield and nitrogen use efficiency in hybrid and conventional barley. *Front. Plant Sci.* 8:1733 10.3389/fpls.2017.01733 PMC5641326 29067032

[B29] Lin T. Y. Maire M. Belongie S. Bourdev L. Girshick R. Hays J. (2014). “Microsoft COCO: common objects in context,” *Computer Vision – ECCV 2014*, eds Fleet D. Pajdla T. Schiele B. Tuytelaars T. (Cham: Springer), 740–755.

[B30] Mingoas K. J. P. Awah-Ndukum J. Dakyang H. Zoli P. A. (2017). Effects of body conformation and udder morphology on milk yield of zebu cows in North region of Cameroon. *Vet. World* 10 901–905. 10.14202/vetworld.2017.901-905 28919680 PMC5591476

[B31] Ouellet V. Vasseur E. Heuwieser W. Burfeind O. Maldague X. Charbonneau É (2016). Evaluation of calving indicators measured by automated monitoring devices to predict the onset of calving in Holstein dairy cows. *J. Dairy Sci.* 99 1539–1548. 10.3168/jds.2015-10057 26686716

[B32] Palombi C. Paolucci M. Stradaioli G. Corubolo M. Pascolo P. B. Monaci M. (2013). Evaluation of remote monitoring of parturition in dairy cattle as a new tool for calving management. *BMC Vet. Res.* 9:191 10.1186/1746-6148-9-191 PMC3850716 24079910

[B33] Pedregosa F. Varoquaux G. Gramfort A. Michel V. Thirion B. Grisel O. (2011). Scikit-learn: machine learning in python. *J. Mach. Learn. Res.* 12 2825–2830.

[B34] Perez P. de los Campos G. (2014). Genome-wide regression and prediction with the BGLR statistical package. *Genetics* 198 483–495. 10.1534/genetics.114.164442 25009151 PMC4196607

[B35] Pritchard T. Coffey M. Mrode R. Wall E. (2013). Genetic parameters for production, health, fertility and longevity traits in dairy cows. *Animal* 7 34–46. 10.1017/S1751731112001401 23031504

[B36] R Core Team (2013). *R: A Language and Environment for Statistical Computing.* Vienna: R Foundation for Statistical Computing.

[B37] Rahaman M. M. Chen D. Gillani Z. Klukas C. Chen M. (2015). Advanced phenotyping and phenotype data analysis for the study of plant growth and development. *Front. Plant Sci.* 6:619 10.3389/fpls.2015.00619 PMC4530591 26322060

[B38] Ren S. He K. Girshick R. Sun J. (2015). Faster R-CNN: towards real-time object detection with region proposal networks. *NeuralPS* 39 91–99. 10.1109/TPAMI.2016.2577031 27295650

[B39] Richardson L. (2007). *Beautiful Soup Documentation.* Available online at: http://citebay.com/how-to-cite/beautiful-soup/

[B40] Rosero A. Granada L. Pérez J. L. Rosero D. Burgos-Paz W. Martínez R. (2019). Morphometric and colurimetric tools to dissect morphological diversity: an application in sweet potato. *Genet. Resour. Crop Evol.* 66 1257–1278.

[B41] Sawa A. Bogucki M. Krêñel-Czopek S. Neja W. (2013). Relationship between conformational traits and lifetime production efficiency of cows. *ISRN Vet. Sci.* 2013:124690. 10.1155/2013/124690 PMC3710600 23878743

[B42] Sogstad ÅM. Østerås O. Fjeldaas T. (2006). Bovine claw and limb disorders related to reproductive performance and production diseases. *J. Dairy Sci.* 89 2519–2528. 10.3168/jds.S0022-0302(06)72327-X 16772570

[B43] Song X. Bokkers E. A. M. van der Tol P. P. J. Groot Koerkamp P. W. G. van Mourik S. (2018). Automated body weight prediction of dairy cows using 3-dimensional vision. *J. Dairy Sci.* 101 4448–4459. 10.3168/jds.2017-13094 29477535

[B44] Tardieu F. Cabrera-Bosquet L. Pridmore T. Bennett M. (2017). Plant phenomics, from sensors to knowledge. *Curr. Biol.* 27 R770–R783. 10.1016/j.cub.2017.05.055 28787611

[B45] Van Hertem T. Alchanatis V. Antler A. Maltz E. Halachmi I. Schlageter-Tello A. (2013). Comparison of segmentation algorithms for cow contour extraction from natural barn background in side-view images. *Comput. Electron. Agr.* 91 65–74.

[B46] van Rossum G. (1995). *Centrum voor Wiskunde en Informatica.* Python tutorial, technical report CS-R9526. Amsterdam: University of Amsterdam.

[B47] Wiggans G. R. Cole J. B. Hubbard S. M. Sonstegard T. S. (2017). Genomic selection in dairy cattle: the USDA experience. *Annu. Rev. Anim. Biosci.* 5 309–327. 10.1146/annurev-animal-021815-111422 27860491

[B48] Yin T. König S. (2019). Genome-wide associations and detection of potential candidate genes for direct genetic and maternal genetic effects influencing dairy cattle body weight at different ages. *Genet. Select. Evol.* 51:4 10.1186/s12711-018-0444-4 PMC6366057 30727969

[B49] Zehner N. Niederhauser J. J. Schick M. Umstatter C. (2019). Development and validation of a predictive model for calving time based on sensor measurements of ingestive behavior in dairy cows. *Comput. Electron. Agric.* 161 62–71.

[B50] Zhang S. Huang W. Zhang C. (2018). Three-channel convolutional neural networks for vegetable leaf disease recognition. *Cogn. Syst. Res.* 53 31–41.

