# Supplementary figures and images for: Estimating Conformational Traits in Dairy Cattle With DeepAPS: A Two-Step Deep Learning Automated Phenotyping and Segmentation Approach
Source: Front Genet. 2020 May 21;11:513. doi: 10.3389/fgene.2020.00513 (PMC7253626; doi:10.3389/fgene.2020.00513)

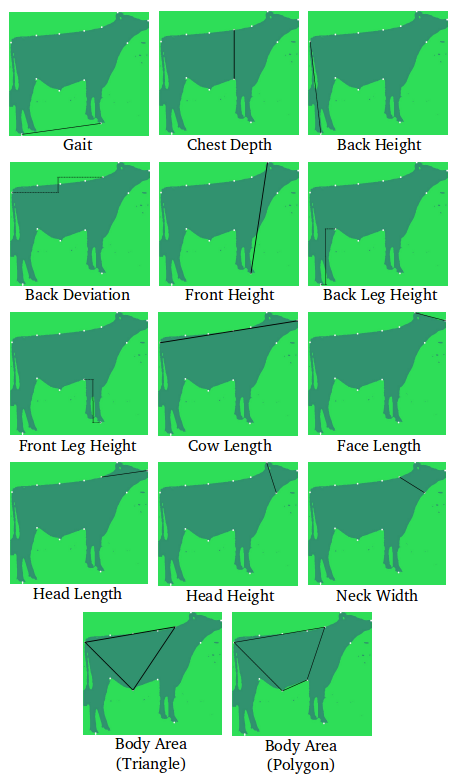

Supplement: FIGURE S1 — Description of the 14 extracted conformational traits. [file Image_1.TIFF]

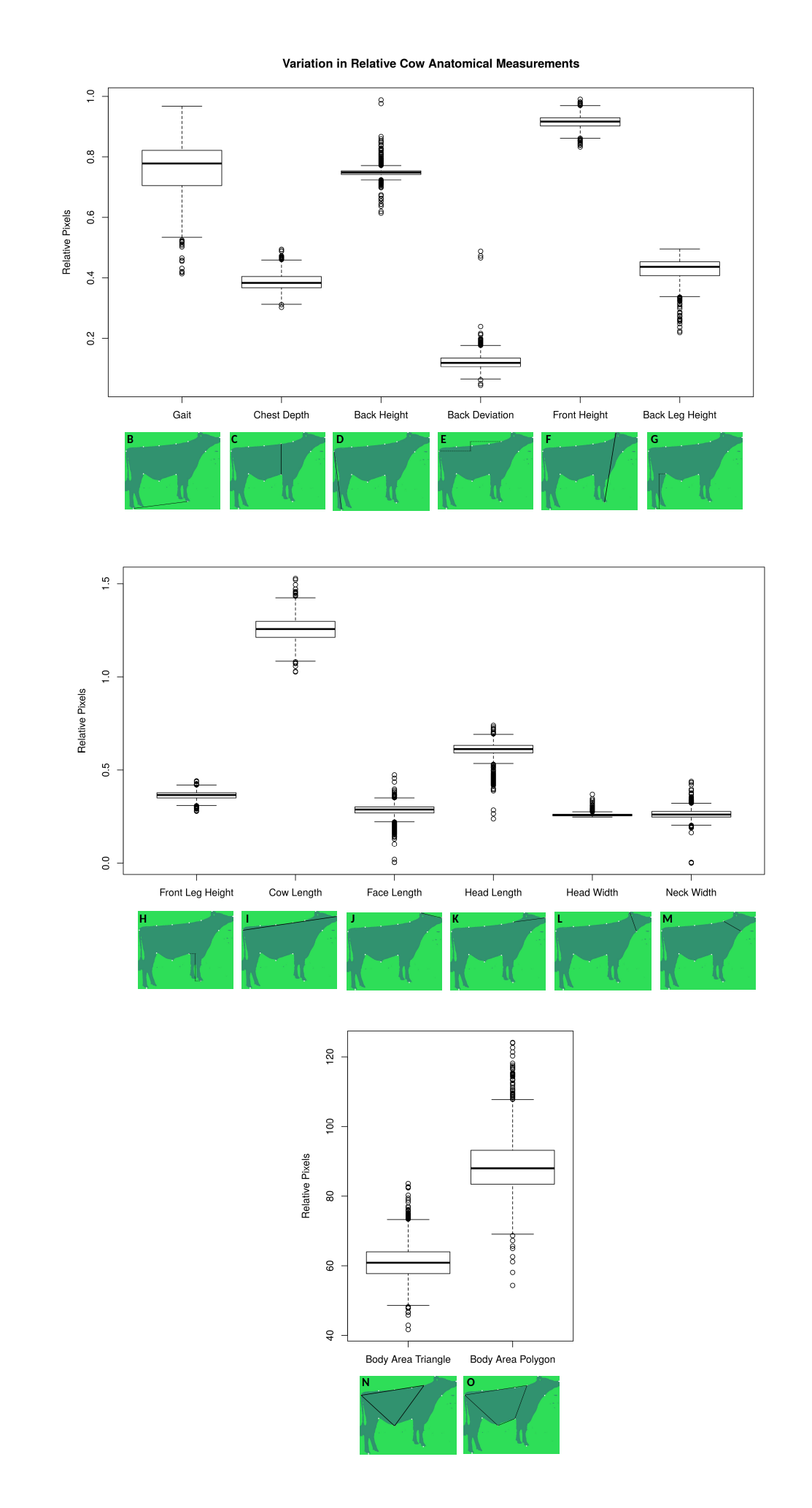

Supplement: FIGURE S2 — Phenotypic distributions for the 14 measured anatomical features (N = 1,062). [file Image_2.TIFF]
